# The Chinese Medicine, Jiedu Recipe, Inhibits the Epithelial Mesenchymal Transition of Hepatocellular Carcinoma via the Regulation of Smad2/3 Dependent and Independent Pathways

**DOI:** 10.1155/2018/5629304

**Published:** 2018-08-08

**Authors:** Shufang Liang, Yong Zou, Jingdong Gao, Xiaolin Liu, Wanfu Lin, Zifei Yin, Juan Du, Ya'ni Zhang, Qunwei Chen, Shu Li, Binbin Cheng, Changquan Ling

**Affiliations:** ^1^Department of Traditional Chinese Medicine, Changhai Hospital, Second Military Medical University, Shanghai 200433, China; ^2^Department of Oncology, Suzhou Hospital of Traditional Chinese Medicine, Suzhou, Jiangsu 215009, China; ^3^Department of Oncology, Zhejiang Provincial Hospital of Traditional Chinese Medicine, Zhejiang 310006, China; ^4^Department of Gastroenterology, Baoshan Branch, Shuguang Hospital Affiliated to Shanghai University of Traditional Chinese Medicine, Shanghai 201900, China

## Abstract

Hepatocellular carcinoma (HCC) is one of the most common malignant tumors worldwide. In China, traditional Chinese herb medicine has been widely used in the treatment of HCC. Jiedu Recipe (JR) is a common used prescription which has shown good results against HCC. However, the exact mechanisms of JR are still unknown. Therefore, we investigated the efficacy of JR on HCC in the current study. JR inhibited the cell viability of both SMMC-7721 and Huh7 cells in both time- and dose-dependent manners. Transwell assay revealed that JR decreased the number of migrated cells of SMMC-7721 cells. JR treatment increased the E-cadherin expression level and decreased the levels of p-Smad2/3 and Smad2/3. Further study showed that JR reversed the effect of TGF*β*1 on the expression of E-cadherin, vimentin, N-cadherin, and MMP2/9. JR also significantly inhibited TGF*β*1-induced migration and invasion of SMMC-7721 and Huh7 cells determined by wound healing assay and transwell assay. TGF*β*1 treatment increased the phosphorylation of Smad2/3, p38 MAPK, JNK, ERK1/2, and Akt in SMMC-7721 cells and pretreatment with JR blocked TGF*β*1-induced activation of Smad2/3 and Akt and MAPKs. In conclusion, JR inhibits liver cancer cells migration and invasion through epithelial mesenchymal transition (EMT) inhibition via Smad2/3 dependent and independent pathways, suggesting it is an effective therapeutic strategy against HCC metastasis.

## 1. Introduction

Hepatocellular carcinoma (HCC), a leading cause of cancer-associated death, is one of the most common malignant tumors worldwide, especially in China. The most common causes of mortality in HCC patients have been identified as cancer recurrence, metastasis, and deterioration of original tumors. Currently, tumor resection is still the main therapeutic method for the treatment of HCC. However, many patients are diagnosed at an advanced stage and thereby lost the operation chance. In addition, the high risk of metastasis is still the main cause for the treat failure and poor prognosis of HCC patients, even those with resectable small tumor [1]. Although many new targeted drugs have been developed in recent years, such as sorafenib, the overall survival is not satisfactory [2]. Furthermore, the targeted drugs are usually too expensive for most of patients.

In China, the herb medicine has been widely used in the treatment of HCC [3–5]. In our institute, we have a continuous interest in the treatment of HCC with Chinese herbs [6, 7]. Jiedu Recipe (JR), composed of valvate actinidia root, salvia chinensis, pseudobulbus cremastrae seu pleiones, and endothelium corneum gigeriae galli, has been utilized for the treatment of HCC and preventing tumor recurrence in postoperative HCC patients for more than a decade [7]. A multicenter, open-label, randomized, controlled study showed that traditional herbal medicine regimen based on JR could efficiently prolong the recurrence-free survival (RFS) of HCC patients after tumor resection [8]. Chen et al. [9] showed that JR prolongs survival of patients with advanced HCC. However, the underlying mechanisms of JR in the treatment of HCC have not been investigated. Therefore, we investigated the role of JR in the proliferation and metastasis of HCC in this study.

## 2. Materials and Methods

### 2.1. Preparation of the JR Extract

The JR consists of valvate actinidia root, salvia chinensis, pseudobulbus cremastrae seu pleiones, and endothelium corneum gigeriae galli. The proportions (w/w) of each herb are as follows: valvate actinidia root 35.7%, salvia chinensis 35.7%, pseudobulbus cremastrae seu pleiones 14.3%, and endothelium corneum gigeriae galli 14.3%. The ethanol extract was prepared as follows: the dried and pulverized herbs were mixed together, and each batch was extracted twice with 85% ethanol by heat under reflux. The ethanol extract was combined, filtrated, and vacuum concentrated at 50°C. Then the extraction was freeze-dried (cold trap temperature: -56°C). Finally, the lyophilized powder was obtained and stored at 4°C before application in experiments. The lyophilized powder of JR was prepared by Shanghai Winherb Medical Technology Co., Ltd. (Shanghai, China). A 50 mg/mL sterilized “JR” was prepared with cell culture medium by two filtrations with 0.22 *μ*m filter. The fingerprinting of JR was determined by high-performance liquid chromatography (HPLC) in the School of Pharmacy, Second Military Medical University for the quality control.

### 2.2. Cell Culture

The human HCC cell lines, SMMC-7721 and Huh7 were cultured in DMEM supplemented with 10% fetal bovine serum (FBS), 100 units/ml penicillin, and 0.1 mg/ml streptomycin (all Hyclone, Life Sciences, Logan, UT, USA) at 37°C in a humidified atmosphere of 5% CO_2_. Cultures were passaged every 2–3 days after reaching 80% confluence. Cells in the logarithmic growth phase were used in the experiment.

### 2.3. MTT Assay for Cell Viability

The SMMC-7721 and Huh7 cells were plated in 96-well culture plates at 1×10^4^ cells per well. After 24 h, various concentrations of JR were added into each well. Subsequent to 24 h or 48 h, MTT assays were performed. MTT was added into each well and incubated for 4 h at 37°C. The medium was then removed and 100 *μ*l DMSO was added into each well. The absorbance at 490 nm was measured by a microplate reader. The mean cell viability was calculated from the absorbance units.

### 2.4. Cell Migration and Invasion Assays

We assayed the invasion and migration activity of cells using a transwell cell culture chamber as described previously [10]. Briefly, SMMC-7721 and Huh7 cells were seeded into the upper compartment of a Transwell Boyden chamber (BD Biosciences, Franklin Lakes, NJ, USA) at a density of 1 ×10^4^/well, with 100 *μ*l serum-free media added into the upper compartment and 500 *μ*l complete media added into the lower compartment. For the invasion assays, the Matrigel (BD Biosciences, Franklin Lakes, NJ, USA) was mixed with serum-free medium at a proportion of 1:5, then applied to the upper compartment of the Transwell Boyden chamber, 100 *μ*l/well, and incubated for 1 h before seeding cells. TGF*β*1 with or without indicated concentrations of JR was added into the upper compartment. After incubation at 37°C for 24 h, images of the cells of each group that had migrated to the chamber of the poly carbon membrane were captured and the results quantified.

### 2.5. Wound Healing Assay

The SMMC-7221 and Huh7 cells (2 × 10^6^ cells/ml) were seeded into 24-well plates with 2-well Ibidi culture-inserts (Ibidi, Munich, Germany). After incubation for 24 h, the culture-inserts were removed carefully. The wounds were observed and their widths were determined using an inverted microscope. The cells were then treated with TGF*β*1 with or without JR for 48 h. The wound width was measured every 24 h.

### 2.6. Real Time RT-PCR

Total RNA was isolated from SMMC-7721 cells with TRIzol reagent (Invitrogen, Carlsbad, CA, USA) as described previously [11]. cDNA was synthesized using a first strand cDNA synthesis kit (Takara Inc., Dalian, P. R. China). Real time PCR was performed using a commercial SYBR Green PCR Master Mix (TOYOBO, Osaka, Japan). The cDNA was amplified under the following conditions: 95°C for 3 min for denaturation and subjected to 40 cycles of 95°C for 10 s, 60°C for 20 s, and 72°C for 25 s. The specific primers are as follows: *β*-Actin, forward: 5′-AGC GGG AAA TCG TGC GTG -3′, reverse: 5′-CAG GGT ACA TGG TGG TGC C-3′; E-cadherin, forward: 5′- CCC AAT ACA TCT CCC TTC ACA G-3; reverse: 5′- CCA CCT CTA AGG CCA TCT TTG -3′; N-cadherin, forward: 5′-CAA GAG GCA GAG ACT TGC GA-3, reverse: 5′-CAC ACT GGC AAA CCT TCA CG-3; Vimentin, forward: 5′- CCT CAC CTG TGA AGT GGA TGC -3, reverse: 5′- CAA CGG CAA AGT TCT CTT CCA-3′. The relative expression level of mRNA in each sample was normalized to its *β*-actin content and was calculated as 2^−ΔΔCt^.

### 2.7. Western Blot Analysis

Total protein from tumor cells was isolated as described previously [12]. The protein concentration was determined by BCA method. Equal quantities of proteins were separated by sodium dodecyl sulfate polyacrylamide gel electrophoresis (SDS-PAGE) and transferred by electroblotting to a nitrocellulose membrane. The membrane was blocked with 5% BSA in TBST buffer (20 mM Tris-HCl, pH 7.4, 150 mM NaCl, and 0.1% Tween 20) overnight at 4°C. Next, the membrane was incubated with specific primary antibodies (Cell Signaling Technology, Danvers, MA, USA; 1:1000) for 2 h and a secondary antibody for 1 h. The signal was visualized with an enhanced chemiluminescence kit (ECL) (Thermo, CA, USA) and imaged with G:BOX Chemi XR5 (Syngene, Frederick, MD, USA).

### 2.8. Statistical Analyses

Data are expressed as means ± SD. One-way analysis of variance (ANOVA) followed by Student-Newman-Keuls tests were used for statistical analysis. Statistical significance was established at P<0.05.

## 3. Results

### 3.1. JR Inhibits the Cell Viability of HCC Cells In Vitro

**Figure 1:**
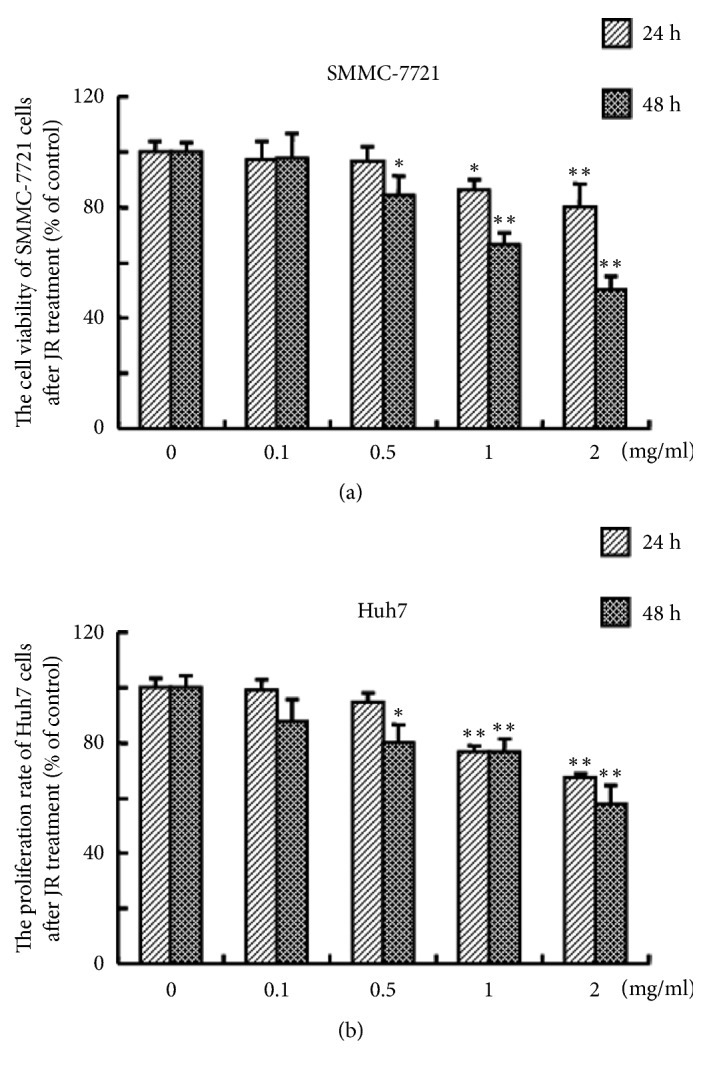
Effect of JR on the cell viability of hepatoma cells. The SMMC-7721 or Huh7 was seeded in the 96-well plates. After incubation for 24 h, indicated concentrations of JR were added into the wells and incubated for 24 or 48 h. Then the cell viability was determined with MTT method. Each bar represents the means ± SD (n=6). ^*∗*^*P*<0.05 and ^*∗∗*^*P*<0.01, compared with control group.

First, we investigated the effect of JR on the viability of liver cancer cells. As shown in Figures 1(a)-1(b), 0.1 mg/ml of JR showed little effect on the viability of both SMMC-7721 and Huh7 hepatoma cells after 24 and 48 h treatment. After treatment with 0.5 mg/ml of JR for 48 h, the cell viability of SMMC-7721 and Huh7 cells significantly decreased compared with control. And the cell viability of SMMC-7721 and Huh7 cells was further decreased with increasing of JR concentration. Therefore, 0.1, 0.5, and 1 mg/ml were used for the next experiments.

### 3.2. JR Inhibits the Migration of HCC Cells

**Figure 2:**
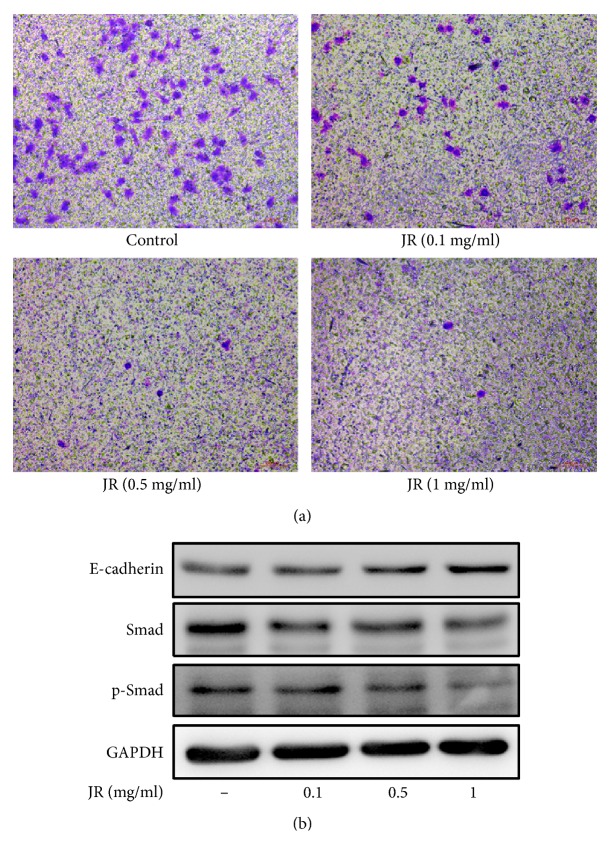
JR inhibits the migration of hepatoma cells. (a) The effect of JR on the migration of SMMC-7721 cells determined by transwell assay. (b) The effect of JR on the expression of E-cadherin and Smad2/3.

Next, we examined the effect of JR on the migration of SMMC-7721 cells. JR (0.1, 0.5, and 1 mg/ml) treatment significantly inhibited the migration of SMMC-7721 cells as determined by transwell assay (Figure 2(a)). Epithelial-cadherin (E-cadherin) is a transmembrane glycoprotein which plays a pivotal role in maintaining cell-cell adhesion and is also a hallmark of epithelial to mesenchymal transition (EMT). Therefore, we determined the levels of E-cadherin in SMMC-7721 cells after JR treatment. Western blot showed that the expression of E-cadherin was increased in SMMC-7721 cells after JR treatment in a dose-dependent manner (Figure 2(b)).

Smad2/3 is an important factor through which the activation signal is received and then transmitted downstream to turn on EMT. We therefore examined the expression of Smad2/3 and phosphorylated Smad2/3 (p-Smad2/3). The expressions of Smad2/3 and the level of p-Smad2/3 were both downregulated after JR treatment in a dose-dependent manner (Figure 2(b)).

### 3.3. JR Inhibits TGF*β*-Induced EMT of HCC Cells

**Figure 3:**
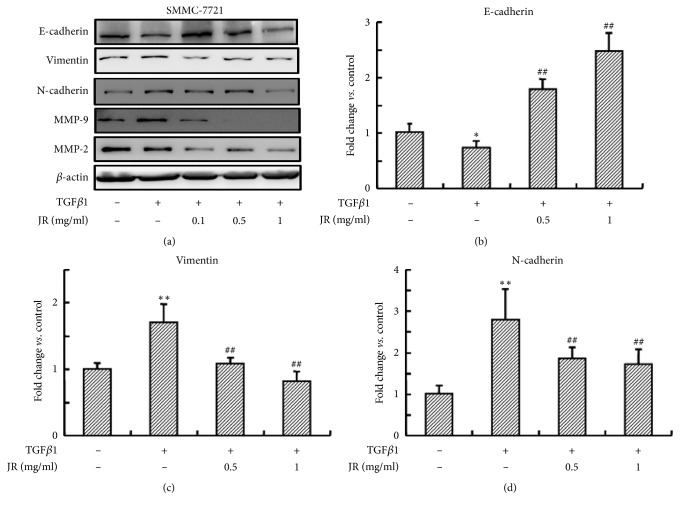
JR inhibits TGF*β*1-induced EMT of HCC cells. (a) Western blot analysis of EMT markers. (b)-(d). The relative mRNA levels of E-cadherin, Vimentin, and N-cadherin. SMMC-7721 cells were seeded into 6-well plate. After 24 h, the cells were pretreated with JR for 2 h, and then stimulated with TGF*β*1 (10 ng/ml) for another 48 h. Each bar represents the means ± SD (n=3). ^*∗*^*P*<0.05 and ^*∗∗*^*P*<0.01, compared with control group; ^##^*P*<0.01, compared with TGF*β*1 group.

Having shown that JR inhibited Smad2/3 and p-Smad2/3 levels in SMMC-7721 cells, we next examined whether JR could suppress TGF*β*1, a Smad2/3 cascade agonist, induced EMT in SMMC-7721 cells. We examined the mRNA and protein expression of the EMT marker genes by real time RT-PCR and western blot. The results in Figure 3(a) showed that TGF*β*1 (10 ng/ml) significantly decreased the expression of E-cadherin and elevated the expression of vimentin, N-cadherin, and MMP2/9 compared with control. Treatment with different concentrations of JR reversed the effects of TGF*β*1. In consistence with western blot results, the changes of the mRNA levels of E-cadherin, N-cadherin, and vimentin induced by TGF*β*1 were also reversed by JR (Figures 3(b)-3(d)).

### 3.4. JR Inhibits TGF*β*1-Induced Migration and Invasion of HCC Cells

**Figure 4:**
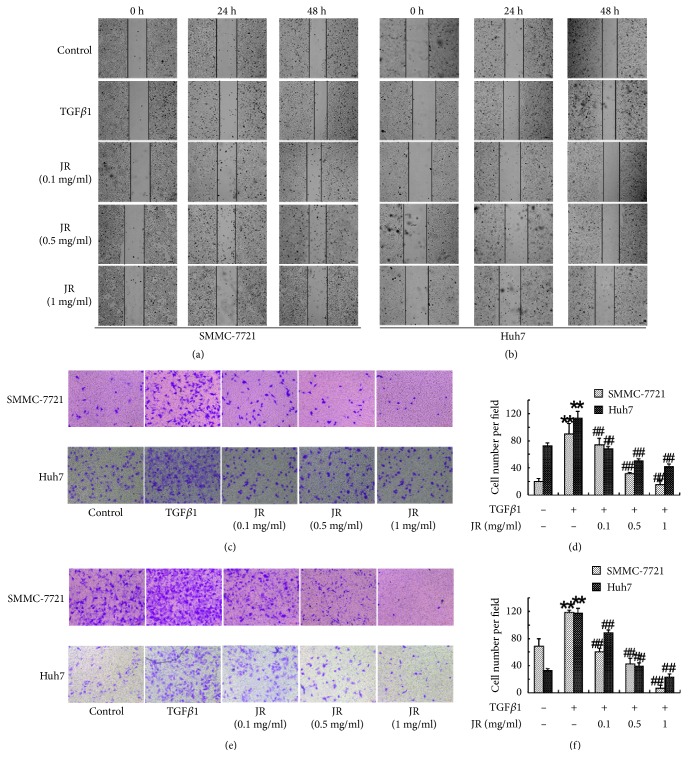
JR inhibits TGF*β*1-induced migration and invasion of HCC cells. The wound healing assay (a-b), and transwell assay for the migration (c-d) and invasion (e-f) of SMMC-7721 and Huh7 cells were performed. Each bar represents the means ± SD (n=3). ^*∗∗*^*P*<0.01, compared with control group; ^##^*P*<0.01, compared with TGF*β*1 group.

TGF*β*1 is able to promote the migration and invasion of cancer cells through the induction of EMT. As shown in Figures 4(a) and 4(b), TGF*β*1 significantly accelerated the wound healing of both SMMC-7721 and Huh7 cells. Different concentrations of JR significantly inhibited TGF*β*1-induced wound healing of SMMC-7721 and Huh7 cells. Transwell assay showed that the TGF*β*1-induced increases in the migration and invasion of SMMC-7721 and Huh7 cells were significantly reversed by JR (Figures 4(c)-4(f)).

### 3.5. JR Inhibits TGF*β*1-Induced Activation of Smad2/3, Akt and MAPKs Pathways

**Figure 5:**
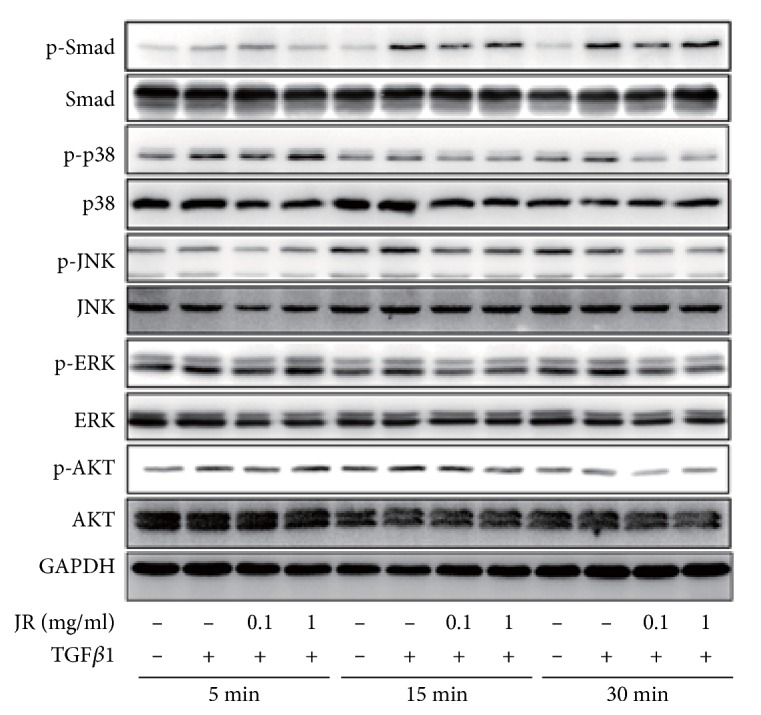
JR inhibits TGF*β*1-induced activation of Smad2/3, Akt and MAPKs pathways. SMMC-7721 cells were seeded into 6-well plate. After 24 h, the cells were pretreated with indicated concentration of JR for 2 h and then stimulated with TGF*β*1 (10 ng/ml) for 5, 15, or 30 min. Then the total protein was extracted and western blot analysis was performed.

TGF*β*1/Smad2/3 cascade signaling plays an important role in the EMT of cancer cells. Therefore, we first determined the effect of JR on TGF*β*1-induced activation of Smad2/3. As shown in Figure 5, TGF*β*1 treatment for 5, 15, and 30 min significantly increased the p-Smad2/3 levels, whereas the total Smad2/3 levels were not obviously altered. JR treatment suppressed TGF*β*1-induced Smad2/3 phosphorylation. Besides TGF*β*1/Smad2/3 cascade, there are also Smad-independent pathways involved into TGF*β*1-induced EMT of cancer, such as MAPKs and Akt. TGF*β*1 treatment also increased the phosphorylation of p38 MAPK, JNK, ERK1/2, and Akt in SMMC-7721 cells. Pretreatment with JR also inhibited TGF*β*1-induced activation of MAPKs and Akt.

## 4. Discussion

In China, herb medicine has been widely used in the treatment of various diseases, including HCC [13]. In the past decade, we showed that JR exerts a surprising effect in preventing the metastasis or recurrence of HCC patients undergone tumor section. In this study, our results showed that JR inhibited the proliferation, migration, and invasion of HCCs in vitro. In addition, JR also inhibited TGF*β*1-induced EMT of HCC cells. Western blots showed that TGF*β*1-triggered activation of Smad2/3, MAPKs and Akt pathways was suppressed by JR pretreatment.

In malignant tumors, the occurrence of EMT usually means tumor migration and invasion. In the EMT process, the loss of cell-cell interaction and polarity, as well as the increase of cell motility, allows tumor cells to escape from the primary location and migrate to distant regions and tissues. E-cadherin, one of the tight junction and adhesion proteins, mediates intercellular adhesion and functions as a key gatekeeper of the epithelial state. The loss of E-cadherin results in the impairment of cell-cell adhesion, which allows detachment of cells and facilitates cancer cell proliferation, invasion, and possibly metastasis. In this study, we first found that JR could inhibit hepatoma cells proliferation and migration. We therefore further observed the expression of E-cadherin in SMMC-7721 cells. The result showed that the expression of E-cadherin was elevated after JR treatment. In addition, the expression of total and phosphorylated Smad2/3 was also downregulated by JR, indicating JR may inhibit EMT in HCC through suppressing Smad2/3 related pathways.

It has been suggested that TGF*β*1 plays a pivotal role in EMT [14]. Up on TGF*β*1 stimulation, TGF*β*1 receptors type I and II form tight complexes leading to phosphorylation of Smad2/3, then leading to the activation of downstream molecules [15]. In the current study, our results showed that JR pretreatment partly blocked TGF*β*1-induced phosphorylation of Smad2/3, indicating JR may inhibit TGF*β*1-induced EMT in hepatoma cells through Smad2/3-dependent pathway. However, although Smad2/3 may be absolutely required for TGF*β*1-induced EMT in malignant tumors, there are also Smad-independent TGF*β*1 pathways involved in EMT [16]. According to previous studies, TGF*β*1 could trigger downstream signaling in EMT depending on MAPKs and PI3K/Akt pathways [17–20]. TGF*β*1 regulates the expression of EMT-related transcription factors through activating the MAPKs and PI3K/Akt pathways, resulting in the loss of E-cadherin [21]. And inhibitors of PI3K, ERK, and p38 MAPK, or agents targeting these pathways, have been used to suppress the development of EMT in various malignant tumors, including HCC [20, 22, 23]. The present study also showed that JR has the ability to inhibit the activation of Akt, ERK, JNK and p38 MAPK, suggesting that JR may suppress TGF*β*1-induced EMT expression and thereby migration and invasion of hepatoma cells via Smad-dependent and Smad-independent pathways.

In conclusion, our study confirmed that JR effectively prevents EMT in hepatoma cells, which provides a reasonable explanation for its inhibitory effects on the migration and invasion of HCC. JR inhibits TGF*β*1 mediated EMT via Smad-dependent and Smad-independent pathways involving the MAPKs and Akt. Our study may provide a new understanding of the mechanisms of JR in the prevention of HCC metastasis and/or recurrence after surgical resection.

## Data Availability

The data used to support the findings of this study are included within the article.

## References

[1] Jia Q., Dong Q., Qin L. (2016). CCN: core regulatory proteins in the microenvironment that affect the metastasis of hepatocellular carcinoma?. *Oncotarget *.

[2] Peng Z., Chen S., Wei M. (2018). Advanced Recurrent Hepatocellular Carcinoma: Treatment with Sorafenib Alone or in Combination with Transarterial Chemoembolization and Radiofrequency Ablation. *Radiology*.

[3] Olugbami J. O., Damoiseaux R., France B. (2017). Atomic force microscopy correlates antimetastatic potentials of HepG2 cell line with its redox/energy status: effects of curcumin and Khaya senegalensis. *Journal of Integrative Medicine*.

[4] Wang X., Wang N., Cheung F., Lao L., Li C., Feng Y. (2015). Chinese medicines for prevention and treatment of human hepatocellular carcinoma: current progress on pharmacological actions and mechanisms. *Journal of Integrative Medicine*.

[5] Zhao G. S., Liu Y., Zhang Q. (2017). Transarterial chemoembolization combined with Huaier granule for the treatment of primary hepatic carcinoma. *Medicine (United States)*.

[6] Lin W., Lu J., Cheng B., Ling C. (2017). Progress in research on the effects of traditional Chinese medicine on the tumor microenvironment. *Journal of Integrative Medicine*.

[7] Yu Y., Lang Q., Chen Z. (2009). The efficacy for unresectable hepatocellular carcinoma may be improved by transcatheter arterial chemoembolization in combination with a traditional Chinese herbal medicine formula. *Cancer*.

[8] Zhai X.-F., Chen Z., Li B. (2013). Traditional herbal medicine in preventing recurrence after resection of small hepatocellular carcinoma: a multicenter randomized controlled trial. *Journal of Chinese Integrative Medicine*.

[9] Chen L. Y., Zhai X. F., Chen Z. (2017). Jie-du granule preparation for the treatment of advanced hepatocellular carcinoma: A retrospective cohort study of 177 patients. *Oncotarget *.

[10] Liu S., Yu M., He Y. (2008). Melittin prevents liver cancer cell metastasis through inhibition of the Rac1-dependent pathway. *Hepatology*.

[11] Zhang Y.-H., Wang Y., Yusufali A. H. (2014). Cytotoxic genes from traditional Chinese medicine inhibit tumor growth both *in vitro* and *in vivo*. *Journal of Integrative Medicine*.

[12] Ghosh S., Sikdar S., Mukherjee A., Khuda-Bukhsh A. R. A. (2015). Evaluation of chemopreventive potentials of ethanolic extract of Ruta graveolens against A375 skin melanoma cells in vitro and induced skin cancer in mice in vivo. *Journal of Integrative Medicine*.

[13] Hu Y., Wang S., Wu X. (2013). Chinese herbal medicine-derived compounds for cancer therapy: a focus on hepatocellular carcinoma. *Journal of Ethnopharmacology*.

[14] Sancisi V., Gandolfi G., Ragazzi M. (2013). Cadherin 6 Is a New RUNX2 Target in TGF-*β* Signalling Pathway. *PLoS ONE*.

[15] Roberts A. B., Tian F., Byfield S. D. (2006). Smad3 is key to TGF-*β*-mediated epithelial-to-mesenchymal transition, fibrosis, tumor suppression and metastasis. *Cytokine & Growth Factor Reviews*.

[16] Derynck R., Zhang Y. E. (2003). Smad-dependent and Smad-independent pathways in TGF-*β* family signalling. *Nature*.

[17] Witte D., Otterbein H., Förster M. (2017). Negative regulation of TGF-*β*1-induced MKK6-p38 and MEK-ERK signalling and epithelial-mesenchymal transition by Rac1b. *Scientific Reports*.

[18] Chen H., Zhou X., Shi Y., Yang J. (2013). Roles of p38 MAPK and JNK in TGF-*β*1-induced Human Alveolar Epithelial to Mesenchymal Transition. *Archives of Medical Research*.

[19] Cho H. J., Baek K. E., Saika S., Jeong M.-J., Yoo J. (2007). Snail is required for transforming growth factor-*β*-induced epithelial-mesenchymal transition by activating PI3 kinase/Akt signal pathway. *Biochemical and Biophysical Research Communications*.

[20] Weng L., Du J., Zhou Q. (2012). Identification of cyclin B1 and Sec62 as biomarkers for recurrence in patients with HBV-related hepatocellular carcinoma after surgical resection. *Molecular Cancer*.

[21] Moustakas A., Heldin C. (2005). Non-Smad TGF-*β* signals. *Journal of Cell Science*.

[22] Yan W., Yu F., Liao J., Liu M., Wang B., Tian D. (2009). S1571 PI3 Kinase/AKT Signaling Mediates Epithelial-Mesenchymal Transition in Hypoxic Hepatocellular Carcinoma Cells. *Gastroenterology*.

[23] Wang H., Zhang C., Xu L. (2016). Bufalin suppresses hepatocellular carcinoma invasion and metastasis by targeting HIF-1*α* via the PI3K/AKT/mTOR pathway. *Oncotarget *.

