# Corrigendum to “The Chinese Medicine, Jiedu Recipe, Inhibits the Epithelial Mesenchymal Transition of Hepatocellular Carcinoma via the Regulation of Smad2/3 Dependent and Independent Pathways”

**DOI:** 10.1155/2022/9780826

**Published:** 2022-05-19

**Authors:** Shufang Liang, Yong Zou, Jingdong Gao, Xiaolin Liu, Wanfu Lin, Zifei Yin, Juan Du, Ya'ni Zhang, Qunwei Chen, Shu Li, Binbin Cheng, Changquan Ling

**Affiliations:** ^1^Department of Traditional Chinese Medicine, Changhai Hospital, Second Military Medical University, Shanghai 200433, China; ^2^Department of Oncology, Suzhou Hospital of Traditional Chinese Medicine, Suzhou 215009, Jiangsu, China; ^3^Department of Oncology, Zhejiang Provincial Hospital of Traditional Chinese Medicine, Hangzhou 310006, Zhejiang, China; ^4^Department of Gastroenterology, Baoshan Branch, Shuguang Hospital Affiliated to Shanghai University of Traditional Chinese Medicine, Shanghai 201900, China

In the article titled “The Chinese Medicine, Jiedu Recipe, Inhibits the Epithelial Mesenchymal Transition of Hepatocellular Carcinoma via the Regulation of Smad2/3 Dependent and Independent Pathways” [1], image concerns were raised on PubPeer [2]. Specifically, in Figure 1(c), panels Huh7 JR (0.1 mg/ml) and Huh7 JR (1 mg/ml) appear to be identical. The authors explained that this was due to an error introduced during manuscript preparation and provided the raw data upon request. The corrected image is given as follows.

## Figures and Tables

**Figure 1:**
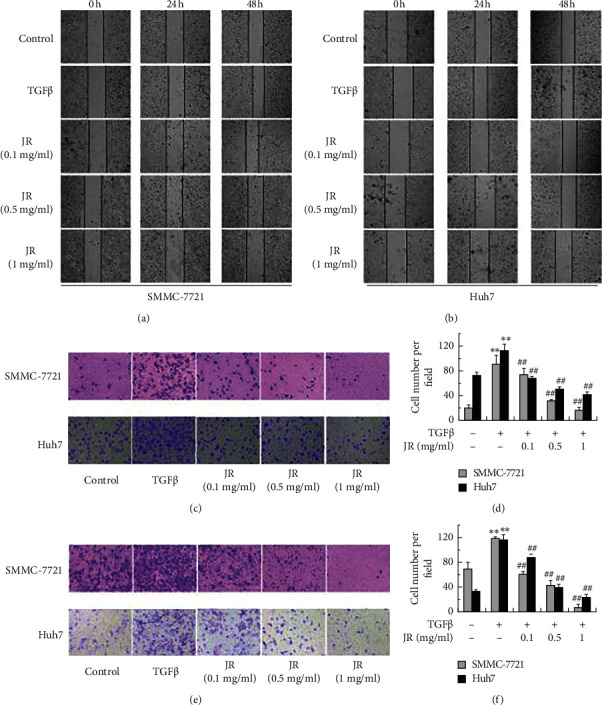
JR inhibits TGFβ1-induced migration and invasion of HCC cells. The wound healing assay (a-b), transwell assay for the migration (c-d), and invasion (e-f) of SMMC-7721 and Huh7 cells were performed. Each bar represents the means ± SD (*n* = 3). ^∗∗^*P* < 0.01, compared with control group; ^##^*P* < 0.01, compared with TGFβ1 group.
